# A Practical Treatise of Dental Medicine, &c.

**Published:** 1853-02

**Authors:** 


*A Practical Treatise of Dental Medicine, &c.* By Thos. E. Bond, A. M., M. D., Prof. of Special Pathology and Therapeutics in the Baltimore College of Dental Surgery. Second Edition. Philadelphia: Lindsay & Blakiston.

This is a well printed octavo of 360 pages. It treats somewhat extensively of matters connected with the practice of dentistry, and notices some subjects not strictly belonging to that department. In his chapter on the use of anæsthetic agents, the author very properly comes to the conclusion that the inconvenience and danger attendant on the use of these substances are too great to justify the dentist in their habitual use. The risk of producing two or three days' discomfort, and even of destroying life, should not be incurred to avoid the comparatively slight and transient pain of dental operations.

The book is for sale in Buffalo by T. Butler.

